# In Search of the Perfect Composite Material—A Chemoinformatics Approach Towards the Easier Handling of Dental Materials

**DOI:** 10.3390/ijms26178283

**Published:** 2025-08-26

**Authors:** Joachim Eichenlaub, Karol Baran, Kamil Urbański, Marlena Robakowska, Jolanta Kalinowska, Bogna Racka-Pilszak, Adam Kloskowski

**Affiliations:** 1 Department of Physical Chemistry, Faculty of Chemistry, Gdansk University of Technology, Narutowicza 11/12, 80-233 Gdansk, Polandadam.kloskowski@pg.edu.pl (A.K.); 2 Department of Dental Prosthetics, Medical University of Gdansk, Elizy Orzeszkowej 18, 80-208 Gdansk, Poland; 3 Division of Orthodontics, Faculty of Medicine, Medical University of Gdansk, Aleja Zwycięstwa 42c, 80-210 Gdansk, Polandjolanta.kalinowska@gumed.edu.pl (J.K.); bogna.racka-pilszak@gumed.edu.pl (B.R.-P.); 4 Department of Public Health and Social Medicine, Medical University of Gdansk, Debinki 7, 80-210 Gdansk, Poland; marlena.robakowska@gumed.edu.pl

**Keywords:** dentistry, QSPR, dental resins, mathematical modeling

## Abstract

Modern dentistry depends on polymer composite materials for a wide range of applications. These materials, mainly composed of polymer resins and reinforced with inorganic fillers, offer mechanical strength, wear resistance, and durability for restorations and prosthetics. This study concentrated on the density and surface tension of monomers often used in dental resins and employed Quantitative Structure–Property Relationship (QSPR) modeling to investigate the influence of monomers’ structural features on these properties. Two main and two auxiliary models to predict both density and surface tension were built and validated. Additionally, two models based on CircuS descriptors were built and analyzed. Molecular descriptors from the models were interpreted and structural characteristics of dental monomers influencing their physicochemical properties were identified. It was found that the presence of heteroatoms increases both of the analyzed properties, while all of the other identified structural features exert an opposite influence on density and surface tension. Furthermore, the study showed that the density of dental monomers can be reliably predicted using the database containing regular organic compounds, but the surface tension requires the database containing specific monomers in order to perform satisfactorily.

## 1. Introduction

Modern dentistry relies heavily on polymer composite materials in a wide range of treatment protocols. These materials are utilized for the reconstruction of tooth structure following damage or decay, both in direct restorations and the cementation of indirectly fabricated prosthetic restorations. They are also employed in the field of orthodontics, securing the orthodontic appliance components to the surfaces of the tooth [1,2].

Dental polymer materials are complex composites designed to meet the mechanical, aesthetic, and biocompatibility requirements of restorative dentistry. Modern materials are usually based on the cross-linking dimethacrylate resins, such as bisphenol A-glycidyl methacrylate (Bis-GMA), which provides the material’s structural framework. In order to tune the physicochemical properties of the matrices, additional monomers are introduced [3]. The final organic matrix consists of a mix of monomers and is reinforced with the inorganic filler particles, such as silica or zirconia, to enhance their mechanical strength, wear resistance, and optical properties [4]. Several other additives, which include coupling agents to ensure strong adhesion, initiators and accelerators to facilitate polymerization, pigments for color matching, and stabilizers to improve the material’s longevity, are also present [2]. The physicochemical properties of the composite material depend on all its components and their precise balance in the final product, which is critical to achieving the desired performance characteristics and ensuring effectiveness in clinical applications [5].

Dental restorative materials should possess the physicochemical properties closely resembling those of natural hard tooth tissues to ensure the compatibility and longevity of the filling [6]. The density of the material, which influences its mechanical properties, is determined by the composition of the resin monomers and inorganic fillers [5]. It should ideally align with that of human enamel, which ranges from 2.84 to 3.00 g/cm^3^, to provide a similar wear resistance and hardness [7]. Further, the density may be correlated with the elastic modulus, with the latter increasing with the increase of the former [8]. A higher elastic modulus in the polymer causes higher contraction stress, leading to microleakage and eventual restoration failure [9,10]. Additionally, composite materials with lower density were found to be better performing by the computer-aided study conducted by Yadav et al. [11]. Even more important is the tension of compositions used in liquid state (adhesives/composites prior to their crosslinking [12,13,14] or liquid solutions intended for different endodontic irrigation [15]), which influences dental materials’ ability to wet and spread across the prepared tooth surfaces. Low surface tension ensures that the material spreads uniformly, allowing it to penetrate into dental tubules and microscopic irregularities on the tooth structure, which is crucial in terms of adhesion and, thus, restoration longevity [12,13,14,15].

To meet the requirements of modern dentistry, in our work, we investigate and attempt to improve the properties of commercially available dental polymer materials by analyzing the influence of the resin monomers on their properties. In this study, we used the Quantitative Structure–Property Relationship (QSPR) mathematical modeling method and analyzed the density and surface tension of the most common monomers used in the field of dentistry. The QSPR method of modeling is based on the assumption that the differences in the properties of the molecules can be correlated with the differences in their structures [16]. This modeling utilizes the existing experimental results and pairs them with the mathematical depictions of the molecules—known as a set of calculated molecular descriptors—in order to find the relationship between them [17]. A thorough interpretation of the models and most influential descriptors enables the researchers both to better understand the mechanisms underlying the molecules’ properties and their behavior and to predict the properties of novel compounds without requiring the lengthy and costly experimental testing. Such an approach is especially valuable in the field of material science and drug design [18]. So far, QSPR modeling has not been used extensively in the field of dentistry. Halder et al. predicted the cytotoxicity of dental monomers [19], Opri et al. were focused on their water/octanol coefficients [20], Abedin et al. used QSPR to design photosensitizers for dental adhesives [21], and Tuğut et al. predicted a glass transition temperature of a complex of dibutyl maleate and polymethyl methacrylate used in prosthetics [22]. To the best of our knowledge, this is the first QSPR approach modeling the surface tension and density of dental materials.

For the readers not familiar with QSPR modeling, a short glossary was added in the Appendix A that contains the definitions of some of the terms used in the study.

## 2. Results

### Modeling

The dataset containing solely the dental monomers consisted of 58 compounds and the values for both density and surface tension were available for 36 of them. Therefore, it was decided to build additional auxiliary models based on the data collected from OCHEM database that contained the data of general organic compounds [23] in order to verify whether it was possible to reliably predict the analyzed physicochemical properties using a significantly larger and widely available database, as it was assumed that the two datasets could occupy the same chemical space. Even though the OCHEM models were only supplementary, it was decided to include them in all of the analyses performed in this study.

In the first step, the analysis of the impact of the molecular representation on models’ performance was carried out. The goal was to establish which of the two options, namely Molecular Fingerprints (MFs) and Molecular Descriptors (MDs), performs the best for studies on the prediction of density and surface tension. Table 1 shows the results of this analysis. It can be seen that two different methods of validation were chosen for the datasets, as the dental monomers sets were too small for a 5-CV and OCHEM sets were too large for LOOCV. It could be observed that for the OCHEM dataset, MF-based density models performed worse than the MD-based ones. On the contrary, while predicting density on the dental monomer dataset, the MF-based model showed the best evaluation metric values. In the case of surface tension models, MD-based models performed better in both datasets. Since the results seemed inconclusive, a comparison with a joint model (incorporating both MFs and MDs−MFs+MDs) was performed. It can be seen that models that used both molecular representations performed quite well in case of every dataset, but in the overall comparison, MD-based models still performed the best in most cases. Additionally, a model based on both MDs and MFs might not necessarily have the best performance. That is the result of a feature selection process in which only a fixed number of descriptors are used in modeling. As a result, the input vector for that model is not a concatenated vector of both representations with doubled size. Even though that approach would guarantee at least the same performance as in the other two modeling methods, it might lead to overfitting. Therefore, MD representation was used for further studies.

Chemical structural features impacting the model’s predictions can depend vaguely on the dataset used for the model’s training. Therefore, a comparative analysis between models trained on OCHEM general chemicals properties and dental monomers datasets was performed. Even though their chemical spaces are quite different, it was assumed that the dental monomers should fit into the applicability domain of the model based on typical organic compounds.

To further investigate the hypothesis, a detailed analysis of chemical diversity was conducted for the two properties under study. The Tanimoto similarity coefficient, a widely used metric for assessing the similarity between compounds, was employed in this analysis. This coefficient can be calculated in two distinct scenarios: within-group and between-group. The within-group scenario refers to the average similarity between pairs of compounds within the same group, whereas the between-group scenario involves pairs of compounds drawn from different groups. In this study, the two groups of interest were small organic compounds and dental monomers.

The within-group analysis of dental monomers revealed a high average similarity of 0.40, indicating a relatively low chemical diversity within this group. In contrast, the average similarity within the dataset of small organic molecules was significantly lower, with values of 0.11 for density and 0.12 for surface tension. These results suggest that the dental monomers exhibit a relatively narrow chemical diversity.

Furthermore, the between-group analysis revealed average similarities of 0.12 between dental monomers and the density dataset, and 0.13 between dental monomers and the surface tension dataset. These findings imply that dental monomers are likely to form distinct clusters in the chemical space when compared to general organic chemical datasets. However, when considering chemical similarity as a measure of variance in chemical structure, dental monomers are not expected to be significantly distinct from typical organic molecules. In fact, the similarity between dental monomers and typical organic molecules is comparable to the similarity among typical organic molecules themselves, indicating that dental monomers do not occupy a unique position in the chemical space.

**Figure 1:**
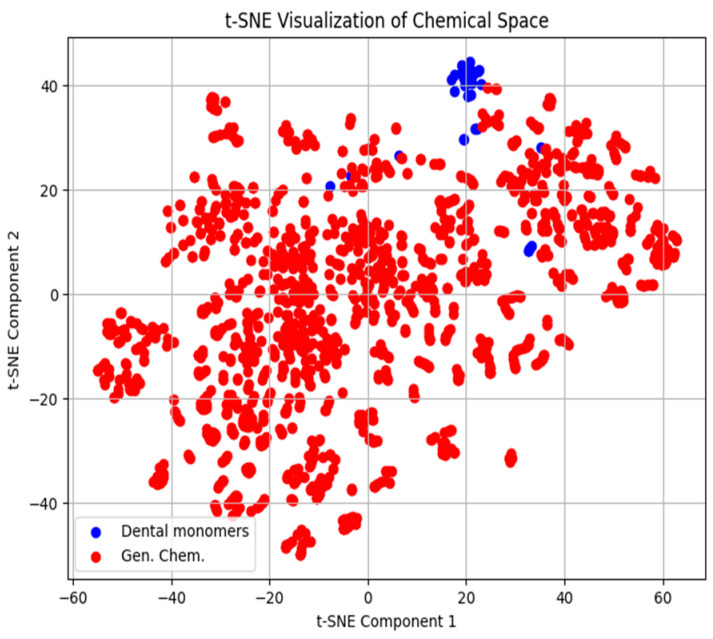
t-SNE plot of surface tension of 30 dental monomers (blue) and 1431 OCHEM compounds (red). It can be seen that the monomers are at the boundary of main cluster, which suggests that this class of compounds may not be approximated by the general chemical compound dataset.

**Figure 2:**
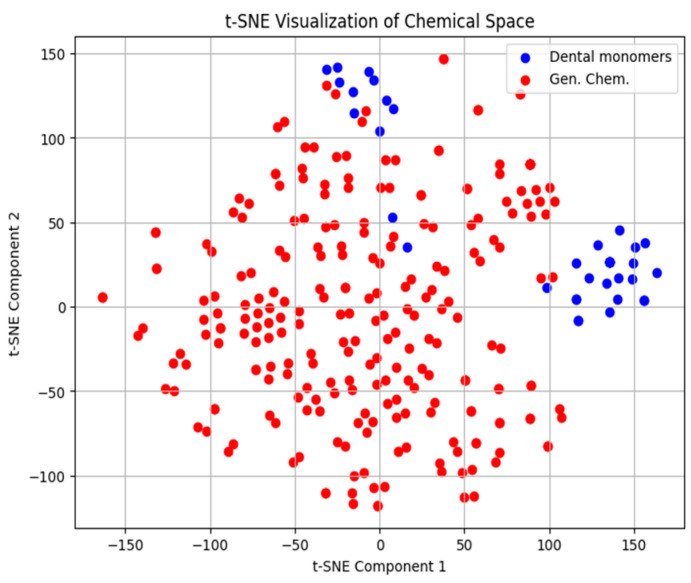
t-SNE plot of density of 30 dental monomers (blue) and 243 OCHEM compounds (red). Similarly to the surface tension, the monomers are mostly at the boundary of the main cluster, yielding the same conclusions.

This observation is further confirmed with t-SNE visualization as shown in Figure 1 and Figure 2. Analysis of the t-SNE plots reveals that the monomers in question occupy a peripheral position within the chemical space, situated at the boundary of the main cluster. Although they do not form a distinct cluster, their proximity to the main group indicates some degree of similarity with the other monomers. However, their relative distance from the central cluster suggests that they may exhibit unique behavior, warranting separate consideration in modeling efforts.

Additionally, another two models were built on OCHEM and dental monomer datasets for training and validated on the dental monomers dataset (both purely experimental and augmented with simulated data) using leave-one-out cross-validation. Their statistical parameters are shown in Table 2. Cross-validation was performed only for dental monomers. OCHEM sets were used as a training auxiliary and these data were not taken into account for the metrics. The reported metrics were calculated as an average of metrics on dental monomers validation sets. It was checked whether adding OCHEM data to the training data would improve the quality of the metrics. This outcome was successfully achieved only for the density model. In this variant, OCHEM data were treated only as an addition to the training set and were not taken into account in cross-validation. Table 2 presents the best variant-the one that gave the highest metric values. The density model performed well in both cases, while the surface tension models performed poorly. The results once again confirm that the surface tension of dental monomers is dependent on the structural features specific only to them.

**Figure 3:**
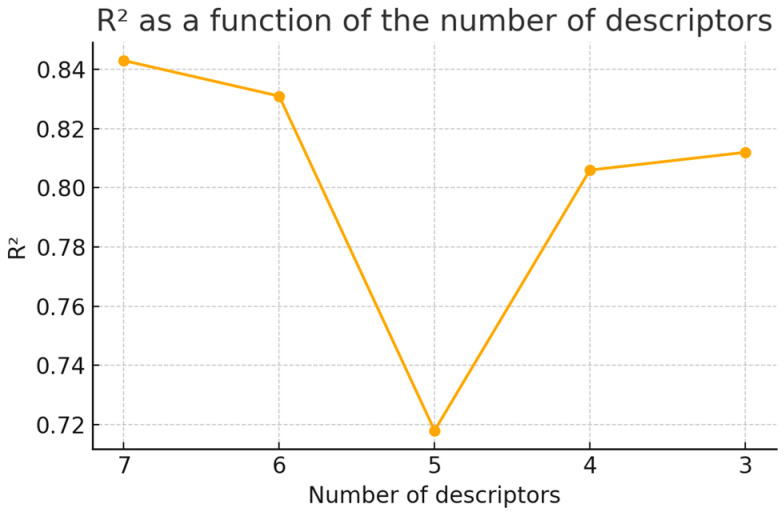
R^2^ of dental monomers models in relation to the number of parameters. Models were constructed using only molecular descriptors. Similar behavior was observed for other models.

The final descriptors of the four models (two MD-based density and two MD-based surface tension models based on dental monomers dataset or OCHEM datasets), along with the permutation analysis [24,25] results, are shown in the Table 3. Permutation importance describes how the changes in the descriptor value influence the model predictions, while Random Forest (RF) importance concentrates on how often the feature is used in determining the model. While the curse of dimensionality describes a continuous challenge, practical performance degradation due to increasing data sparsity and computational demands often becomes notably pronounced in machine learning models when the number of features exceeds approximately six, necessitating significantly more data for effective modeling. Therefore, six features were used initially for modeling. To validate whether adding more descriptors would lead to better model performance, experiments with other possible descriptor amounts among the input were conducted for the dental monomers dataset, revealing very similar performance in both scenarios. Figure 3 shows the R^2^ of dental monomers models in relation to the number of parameters. It can be seen that decreasing the number of descriptors yields slightly worse statistical parameters of the model, yet still at a satisfactory level. Decreasing the number of descriptors from six to five results in the largest drop in R^2^ value. It is worth noting that the R^2^ value increases again after further reduction in descriptor number. It could be easily observed that some of the features were shared between models for predicting the same property based on different training datasets. For example, both models for predicting density heavily relied on Autocorrelation of a Topological Structure (ATS) descriptors, regardless of the dataset source. However, that observation was no longer valid for models predicting surface tension. This observation once again suggests that the two datasets differed significantly based on the chemical structural features of the compounds in them.

The workflow of the study is available in Electronic Supplementary Information (ESI) as Figure S1. Values of the descriptors used in the models from dental monomer dataset can be found in Table S2 in ESI.

## 3. Discussion

**Figure 4:**
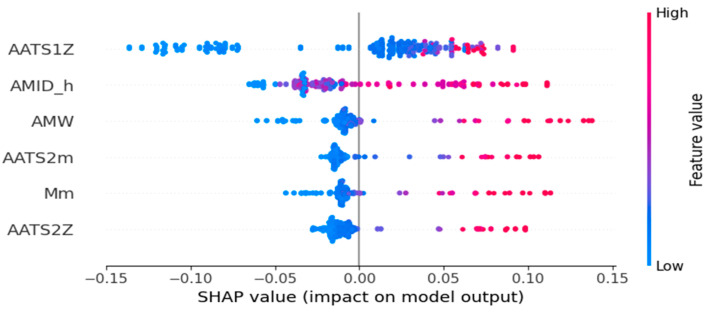
SHAP plot of the density OCHEM model. Dots on the left side of the plot decrease the density and dots on the right side increase its value. The influence of each descriptor is given at the end of its corresponding paragraph.

**Figure 5:**
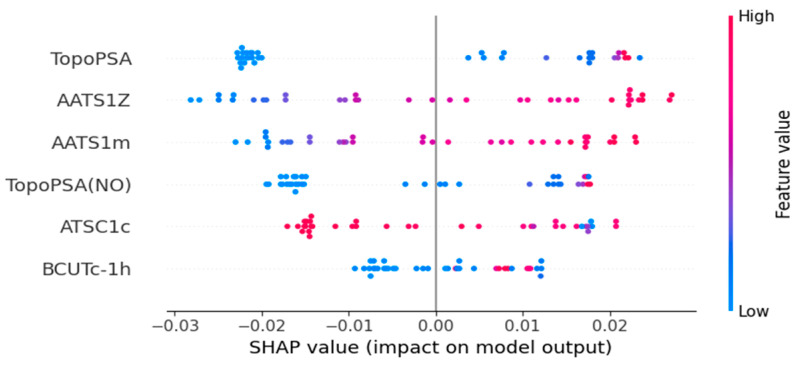
SHAP plot of the density of the dental monomers model. Dots on the left side of the plot decrease the density and dots on the right side increase its value. The influence of each descriptor is given at the end of its corresponding paragraph.

**Figure 6:**
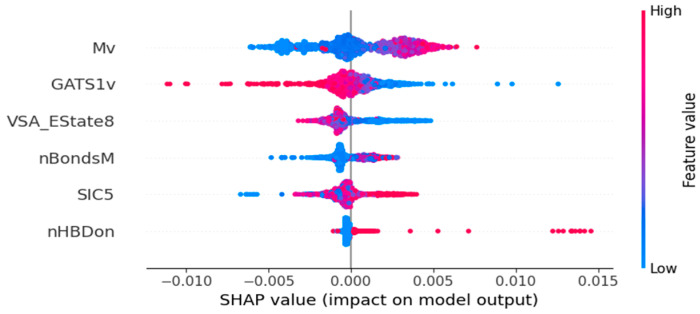
SHAP plot of the surface tension OCHEM model. Dots on the left side of the plot decrease the surface tension and dots on the right side increase its value. The influence of each descriptor is given at the end of its corresponding paragraph.

**Figure 7:**
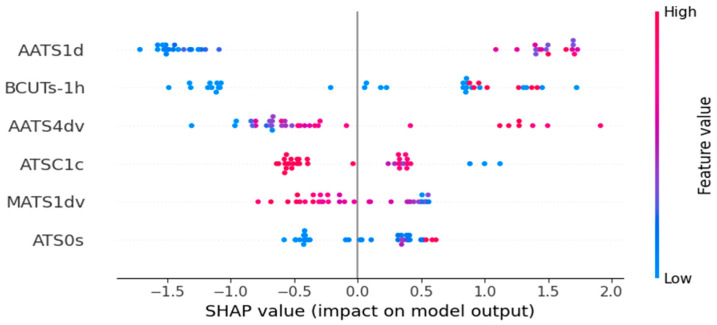
SHAP plot of the surface tension of the dental monomers model. Dots on the left side of the plot decrease the surface tension and dots on the right side increase its value. The influence of each descriptor is given at the end of its corresponding paragraph.

The descriptors used in the four models from the study were calculated from SMILES, therefore they belong solely to 2D categories of molecular descriptors. The following chapter concentrates on the interpretation of the descriptors in the models and the SHAP diagrams shown in Figure 4, Figure 5, Figure 6 and Figure 7. Most of the descriptors used in the study belong to the same category of 2D autocorrelation descriptors, known as Autocorrelation of a Topological Structure (ATS) or Moreau–Broto autocorrelation descriptors. Therefore, to avoid repetition and for ease of reading, the general information about them will be provided in Appendix B and specific descriptors will be discussed afterwards with additional in-depth analysis supplied for both models based on the dental monomers.

### 3.1. Density Model Based on OCHEM Dataset

**AMID_h** is a descriptor defined as the *averaged molecular ID on h atoms* [26]. It belongs to the group of descriptors proposed by Randić in order to provide a unique identification number for individual compounds that could also be used to compare them. Molecular ID numbers are calculated as weighted path counts on the graph representing the molecule [27]. In case of AMID_h descriptor, molecular ID numbers are calculated and averaged only for heteroatoms present in the structure [26]. Higher values of this descriptor increase the density, as shown in Figure 4.

**AATS1Z** is an AATS descriptor, defined as *averaged Moreau–Broto autocorrelation of lag 1 weighted by atomic number*. Therefore, in the case of the dental monomers, its value is highly influenced by the presence of heteroatoms [26]. Higher values of this descriptor increase the density.

**AMW** is one of the most basic molecular descriptors, as it is calculated as the *averaged molecular weight* of the analyzed compound—the molecular weight is divided by the number of atoms in the molecule [26]. Higher values of this descriptor increase the density.

**Mm** is a descriptor that is related to the molecular mass of the analyzed compound. It is described as a *mean of constitutional weighted by mass*. The descriptor belongs to the Constitutional Mean category of molecular descriptors, which is, in general, calculated with Equation (1):

(1) $$Mm=\frac{\sum_{i=1}^{A}\frac{p_{i}}{p_{c}}}{A}$$

where *A* is the number of atoms in the molecule, *p_i_* is a property of an atom, and *p_c_* is the same property of the carbon atom. In this case, the mass of each atom is scaled in relation to the mass of the carbon atom [26]. Higher values of this descriptor increase the density.

**AATS2m** is a descriptor defined as an *averaged Moreau–Broto autocorrelation of lag 2 weighted by mass* [26]. It is another descriptor highly dependent on the mass of the atoms present in the dental monomer molecules, with heteroatoms being the main differentiating factor. Higher values of this descriptor increase the density.

**AATS2Z**, the last of the descriptors in this model, also belongs to the AATS descriptors category. It is the *averaged Moreau–Broto autocorrelation of lag 2 weighted by atomic number* [26]. Higher values of this descriptor increase the density.

### 3.2. Density Model Based on Dental Monomer Dataset

**TopoPSA** is a descriptor determined using a group contribution method. By definition, it is a *topological polar surface area* descriptor, calculated as shown below:

(2) $$TopoPSA=\sum_{i}N_{i}G_{i}$$

In Equation (2), *N_i_* stands for the recurrence of analyzed atom types in the molecule and *G_i_* stands for the value of surface contribution assigned to the specific polar atom types [28,29]. It is worth noting that these values are derived from the mathematical model, not the pure experimental data. However, the statistical parameters of that model were excellent with R^2^ = 0.982, so the predicted values are reliable enough to be used in other QSPR models [26]. The atoms analyzed by this descriptor are nitrogen, oxygen, phosphorus, and sulfur. There are 26 atom types associated with nitrogen, 6 atom types for oxygen, 7 for sulfur, and 4 for phosphorus. This descriptor is mainly used in medicinal chemistry, as it has been proven to be a reliable predictor of the cell permeability of drugs administered to humans [30]. Higher values of this descriptor increase the density, as is shown in Figure 5. In this dataset, higher values of the descriptor are associated with the presence of oxygen and nitrogen atoms. It is worth noting that the -OH groups increase the value to a smaller extent than the sole =O or -O- atoms, and the nitrogen contribution is even smaller. Ethyleneglycol dimethacrylate shows the highest value of this descriptor from the entire dataset and the whole group of alkane-based acrylates and methacrylates, such as butyl acrylate or n-propyl methacrylate, show the lowest values. Increasing the alkyl chain length has no effect on this descriptor.

**AATS1Z** is a descriptor that is also present in the density model based on the OCHEM dataset and the definition has already been provided in that paragraph. Higher values of this descriptor increase the density. A few structural dependencies may be observed in case of this descriptor. Longer alkyl chains decrease its value significantly, as well as the presence of the methacrylate group instead of the acrylate one. In order to increase the value and thus the density, -OH groups should be present in the molecule structure.

**TopoPSA(NO)** is a modification of the TopoPSA descriptor that uses only nitrogen and oxygen atoms in the calculations, omitting other polar atoms present in the molecule. It is also mainly used in medicinal chemistry, as the nitrogen and oxygen polar groups in the molecule are the main factor that influences the solubility in lipids. Therefore, it is possible to model and predict the way the drug is metabolized [30]. Additionally, the PSA values correlate with hydrogen bonding, which is especially important in the case of our research, as the density of organic materials is highly dependent on the presence of hydrogen bonds. Surprisingly, the model with the best statistical parameters contained both TopoPSA and TopoPSA(NO). However, despite being similar, the descriptors have a completely different distribution on the corresponding SHAP plot. Higher values of this descriptor increase the density. The structural indications for this feature are the same as in the case of TopoPSA.

**AATS1m** is a descriptor defined as an *averaged Moreau–Broto autocorrelation of lag 1 weighted by mass* [26]. Interestingly, in the OCHEM model, a very similar descriptor was used, but the descriptor was calculated at lag 2 instead. Higher values of this descriptor increase the density. Terminal -OH groups and the presence of cyclic structures increase this descriptor value. On the other hand, increasing the length of the alkyl chain drastically decreases it, as methyl acrylate and lauryl acrylate are on opposite spectra of this descriptor range. Additionally, acrylate groups instead of methacrylate groups increase the density of monomers.

**ATSC1c** is a descriptor defined as a *centered Moreau–Broto autocorrelation of lag 1, weighted by gasteiger charge*. The gasteiger charge calculation is based on the assumption that atoms interact differently with each other based on their electronegativity. During the computation, a set charge values are assigned to the atoms and are later distributed between the neighboring atoms based on their electronegativity values [26,31]. Therefore, the value of the ATSC1c descriptor is highly dependent on the presence of heteroatoms in the monomers. It is the only descriptor in both density models for which higher values decrease the density. Even though the descriptor is strictly associated with the presence of heteroatoms, in the monomer dataset its value is also dependent on the degree of branching, as the more branched isomers have higher values of this descriptor. In the case of the ATSC1c descriptor, longer alkyl chains increase the density, which is an exception in this dataset.

**BCUTc-1h** belongs to the BCUT category, which consists of descriptors calculated from the Burden matrices and on their own can be used to compare and find similar molecules in larger databases. Burden matrix is created by constructing a connectivity matrix from a hydrogen-depleted molecular graph with diagonal elements corresponding to the analyzed property (classic Burden matrix uses the Z atomic number), off-diagonal elements are dependent on the bond order from the graph with values set to 0.1, 0.2, 0.3 and 0.15 for single, double, triple, and aromatic bonds, respectively. All of the other matrix elements have a fixed 0.001 value and elements corresponding to terminal bonds are increased by 0.01. In order to represent a molecule by a single value, eigenvalues can be calculated from the Burden matrix [32]. The BCUTc-1h descriptor is defined as the *first highest eigenvalue of Burden matrix weighted by gasteiger charge* [26]. In this case, the diagonal elements represent the gasteiger charges that are dependent on the electronegativity and were already discussed beforehand. Higher values of this descriptor increase the density. Two main factors increasing the density in the case of this descriptor are the presence of -OH. The acrylate and methacrylate groups’ relationship is the same as in the two AATS descriptors, as the additional methyl group decreases the density.

### 3.3. Surface Tension Model Based on OCHEM Dataset

**Mv** is another descriptor from the Constitutional Mean category. It is defined as the mean of constitutional weighted by van der Waals volume. It is calculated as shown in Equation (10). Van der Waals volume relates directly to the space that is occupied by the atom [26]. In case of our research, the space taken by the atoms, especially the larger ones, substantially influences the folding of the polymers and their ability to form hydrogen bonds. Consequently, it is crucial in defining the surface tension value of the compound. Higher values of this descriptor increase the surface tension, as shown in Figure 6.

**GATS1v** is another descriptor dependent on the van der Waals volume. It is defined as the *Geary coefficient of lag 1 weighted by van der Waals volume*. Both the importance of van der Waals volume and GATS descriptors were discussed above, thus, no further explanation is needed. Higher values of this descriptor decrease the surface tension.

**VSA_EState8** descriptor calculates two distinct parameters—van der Waals surface area (VSA) and Electrotopological state (E-State). E-state values are then divided into bins based on the VSA contributions. VSA_Estate8 descriptor is defined as *VSA EState Descriptor 8 (6.45 ≤ x < 7.00)*, which means that only the E-state values falling into the 8th bin are being accounted for [26]. Van der Waals surface is calculated based on the predefined atomic van der Waals radii and interatomic bond lengths. E-state is an atomic property incorporating the information from the calculated intrinsic state, but expanding on it, as it also includes the influence of the environment [33]. The intrinsic state is described further in the paragraph concerning BCUTs-1h descriptor. The effect of the environment, described also as a perturbation factor, can be explained as the influence of the electronic field of the entire molecule on the sole analyzed atom. It is a sum of interactions of all the atom pairs in the molecule, inversely proportional to the distance between them. Higher values of this descriptor decrease the surface tension.

**nBondsM** is a simple quantitative descriptor defined as a *number of multiple bonds in non-kekulized structure*. Simply put, kekulized structures have the bond orders assigned to the aromatic bonds, e.g., kekulized benzene ring is represented as three single and three double alternating bonds [34]. Therefore, the nBondsM descriptor counts only the multiple bonds that are unrelated to aromatic structures. Higher values of this descriptor increase the surface tension.

**SIC5** is a descriptor that belongs to the Structural IC category. It is defined as a *5-ordered structural information content*. The descriptor value is dependent on Shannon’s entropy and based on the distribution of the atoms in the molecule into subsets and the probability of finding the analyzed atom in the given subset [26]. Information Content descriptors are not unique for each molecule and may have corresponding values for different molecules, provided their chemical bonds and valence electron distribution are similar. They are also correlated with log P (octanol-water) values and may be utilized to predict the bioactivity of organic molecules [35]. Higher values of this descriptor increase the surface tension.

**nHBDon** is another unsophisticated quantitative descriptor, which is the *number of hydrogen bond donors* [26]. The descriptor counts all the occurrences of possible hydrogen bond donors in the molecule. Higher values of this descriptor increase the surface tension.

### 3.4. Surface Tension Model Based on Dental Monomer Dataset

**AATS1d** is a descriptor defined as an *averaged Moreau–Broto autocorrelation of lag 1 weighted by sigma electrons*. Therefore, it is dependent on the occurrence of bonds between the atoms, but is indifferent to the bond order, as it does not differentiate between single, double, or triple bonds [26]. Higher values of this descriptor increase the surface tension, as shown in Figure 7. The highest value of this descriptor was associated with the presence of cyclic structures and aromatic bonds. Additionally, the presence of methacrylic groups and increasing the alkyl chain length increase the AATS1d value. The opposite effect can be observed for the ethylene glycol groups.

**BCUTs-1h** is a descriptor defined as the *first highest eigenvalue of Burden matrix weighted by intrinsic state* [26]. It belongs to the BCUT category that was discussed in detail in the previous paragraph. In this case, the diagonal elements in the Burden matrix correspond to the intrinsic state of the atoms, the parameter that is dependent on the number of π and lone pair electrons, electronegativity and the degree of branching of the molecule. The higher the number of listed electrons, electronegativity, the higher is the intrinsic state value. On the other hand, it decreases with the increased branching [36,37]. Higher values of this descriptor increase the surface tension. This descriptor is not dependent on the acrylate and methacrylate groups, but increases with the elongation of the alkyl chain and the presence of heteroatoms. It is worth noting that the nitrogen atoms have a greater impact on the descriptor value than the oxygen atoms.

**AATS4dv** is an *averaged Moreau–Broto autocorrelation of lag 4 weighted by valence electrons*. Valence electrons determine the general reactivity of the molecule and the ability to form bonds. As valence electrons take part in the formation of hydrogen bonds that influence the surface tension of molecules, the presence of a descriptor corresponding to their occurrence is expected [26]. Higher values of this descriptor increase the surface tension. The highest values of this descriptor were associated with the monomer molecules with two or more oxygen atoms in close vicinity. It is also dependent on the size of the molecule, as its value increases with the atom count. Additionally, methacrylate groups were found to decrease the descriptor value.

**ATSC1c** is a descriptor that is also present in the density model based on the dental monomers dataset and the definition has already been provided in that paragraph. Higher values of this descriptor decrease the surface tension, which is a common characteristic shared by both density and surface tension models.

**MATS1dv** is defined as a *Moran coefficient of lag 1 weighted by valence electrons*. It is a second descriptor in the model that is dependent on the valence electrons, further proving their importance. Moran coefficient calculation was discussed at the beginning of this paragraph. Higher values of this descriptor decrease the surface tension. Increasing the alkyl chain length and addition of -OH groups increase its value, while cyclic structures, aromatic rings and ethylene glycol groups significantly decrease it. In contrast to the previous two descriptors, homologous methacrylates have a higher value than acrylates.

**ATS0s** is a descriptor defined as *Moreau–Broto autocorrelation of lag 0 weighted by intrinsic state*. Since it is a lag 0 descriptor, it is a sum of squared values of all of the intrinsic states values calculated for the whole molecule [26]. Higher values of this descriptor increase the surface tension. Since it is dependent solely on the sum of all calculated sub-values, it increases with the size of a molecule and the presence of heteroatoms also has a significant positive impact. Methacrylate groups were also associated with higher values of the descriptor, but in this case, it might be correlated simply with the additional carbon atom in the calculations.

### 3.5. Structural Interpretation of the CircuS Models

**Figure 8:**
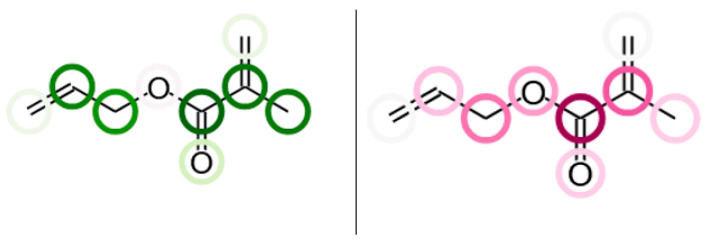
An allyl methacrylate molecule with structural elements associated with increased surface tension (green, on the left) and decreased density (red, on the right).

Several structural dependencies can be observed in the models prepared using CircuS 1.2 software, which is explained in detail in Paragraph 4. The color scheme used in this study is based on the two complementary colors—green and red—that are assigned to positive and negative contributions, respectively. The more intense the color, the larger the contribution. Green circles are associated with molecule fragments whose presence increases the analyzed property value and red circles bear the opposite relationship [38]. In most cases, the molecular fingerprints identified and modelled by the CircuS influence the density and surface tension of dental monomers inversely, which can be observed in the allyl methacrylate molecule, shown in Figure 8. The additional graphical representation of selected monomer structures is available in Table S3 in the ESI. In most structures, there is a visible negative correlation between fragments that increase the surface tension and decrease the density, such as oxygen bridges connecting alkyl chains, ketone groups, or tertiary carbon atoms at the terminal positions in the molecule.

**Figure 9:**
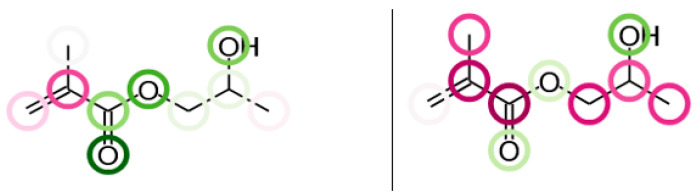
A 2-hydroxypropyl methacrylate molecule with -OH groups associated with increased surface tension (on the left) and increased density (on the right). The difference in the effect of methyl groups is also visible.

Aromatic rings tend to have a greater influence on the surface tension than on the density, which corresponds with the surface tension models that were more dependent on the intrinsic states of the molecules. There are few exceptions of these observed regularities, and the results show that finding the compound that can be characterized with both the analyzed properties at the low values may prove to be an elaborate task. However, among the mutually exclusive dependencies, the presence of some structural elements was found to be positively correlated with both the surface tension and density. In general, the presence of heteroatoms in forms of hydroxyl and amine groups increases the corresponding properties. Additionally, the methyl group in methacrylates tends to have a stronger influence on the density (decreasing it substantially) than on the surface tension. Both of these findings are visualized in the Figure 9, representing a molecule of 2-hydroxypropyl methacrylate. Therefore, it is worthwhile to take into account the presence of such groups in the process of designing new dental materials in terms of the ergonomics of their utilization.

### 3.6. Model Comparison

The QSPR density models are dependent on two main factors—presence of heteroatoms (four descriptors in OCHEM model, five in dental monomers) and mass of the molecule (two of them and one, respectively). Both of those properties are well known to be correlated with the density of chemical compounds. Occurrence of atoms like nitrogen and hydrogen (covalently linked to a very electronegative heteroatom-N, O) is associated with the ability of creating hydrogen bonds between the separate molecules, which is one of the most essential factors in determining many physicochemical properties, as it influences the way the molecules fold and interact in the bulk of the liquid. The results are in accordance with the chemical space analyses’ results carried out before, as they show that the density of the dental monomers and the general organic molecules dataset is dependent on the same structural features. In general, higher density can be associated with the presence of heteroatoms, while the lower values were found for the molecules containing long alkyl chains and methacrylate groups instead of acrylates.

On the other hand, the same relationship cannot be stated between the two chemical groups in the case of the surface tension. Chemical space analyses suggested that the surface tension of the dental monomers may be dependent on their specific and distinct features. As suspected, the surface tension models were based on the more diverse and, in most cases, detailed set of descriptors. The OCHEM model was influenced by the van der Waals volume, number of multiple bonds, hydrogen bond donors, electrotopological state, and, by extension, by the presence of valence electrons, π bonds, and electronegativity. The dental monomers model was dominated by the ATS category descriptors, which covered five out of six in the dataset. The descriptors corresponded to the distribution of multiple bonds, valence electrons, and electronegativity of the atoms in the molecule. Long alkyl chains present in the molecules increase the surface tension, as well as heteroatoms and methacrylate groups instead of acrylates.

## 4. Materials and Methods

### 4.1. Datasets

Two publicly available datasets from the Online Chemical Modeling Environment (OCHEM) were used to train and evaluate the models. OCHEM is a large collection of publicly available datasets for various physical and chemical properties of molecules [23]. To eliminate the impact of temperature and pressure, data were extracted only from experiments conducted at 298.15 K and under 0.1 MPa pressure. After narrowing down the dataset, we acquired an OCHEM database consisting of 243 molecules with density measured in 298.15 K and under 0.1 MPa and values ranging from 0.626 to 1.614 g/cm^3^. A similar database was prepared for surface tension and consisted of 1430 molecules with measured surface tension values, ranging from 9.42 to 66.18 dyn/cm.

The data on dental monomers were collected using ChemSpider (https://www.chemspider.com/, (accessed on 13 July 2025)), based on the dataset collected by Halder et al. [19], which consisted of 58 acrylic acid-based dental monomers. Unfortunately, the available experimental data on the surface tension were very scarce, which is a limitation to our study. Due to the small size of the experimental dataset, it was augmented with simulated data of 33 compounds from reliable and validated QSAR models adopted by ChemSpider. As a result, we obtained 36 dental monomers with both density and surface tension predicted values. A full list of monomers along with values of density and surface tension is available in Table S1 in Electronic Supplementary Information (ESI), where simulated data are visible in columns F and K.

### 4.2. Descriptor Calculation and Model Building

To develop predictive models for properties of interest, a combination of feature engineering, feature selection, and machine learning regression algorithms were employed. Molecules were represented using two main approaches, mainly Molecular Descriptors (MDs) and Molecular Fingerprints (MFs). Molecular descriptors are numerical representations of the structural and chemical properties of molecules, which can be used to capture the underlying relationships between molecular structure and biological activity or physical properties [39]. MDs were calculated using the Mordred 1.2.0 Python package [28]. For simplicity, only 2D descriptors were included. Molecular fingerprints, on the contrary, represent the molecular structure as a binary vector, where each bit corresponds to the presence or absence of a specific substructure or feature [40]. In this study, we used a type of molecular fingerprint (Morgan fingerprint) that encodes the molecular structure as a series of circular substructures of a specified radius. This allows for the capture of both local and global structural features, such as functional groups, rings, and branching patterns [41]. MFs were used in the form of Morgan fingerprints with a radius of 2 and a fingerprint size of 2048 bits. In this study, we employed a combination of molecular fingerprints and 2D descriptors, which provide a comprehensive representation of molecular topology, physicochemical properties, and electronic features. The list of calculated MFs and MDs is available in Table S1 in ESI. To visualize the high-dimensional chemical space of the dataset, the t-distributed Stochastic Neighbor Embedding (t-SNE) was used. It is a non-linear dimensionality reduction technique that maps data to a lower-dimensional space while ensuring that clusters are still visible in lower-dimensional space. Default t-SNE parameters were utilized:

class sklearn.manifold.TSNE(n_components = 2, *, perplexity = 30.0, early_exaggeration = 12.0, learning_rate = ‘auto’, max_iter = None, n_iter_without_progress = 300, min_grad_norm = 1 × 10^−7^, metric = ‘euclidean’, metric_params = None, init = ‘pca’, verbose = 0, random_state = None, method = ‘barnes_hut’, angle = 0.5, n_jobs = None, n_iter = ‘deprecated’)

The resulting t-SNE plots revealed the relationships between molecules in the dataset and identified clusters and patterns that are not immediately apparent in the high-dimensional space [42]. The t-SNE method was used as implemented in the scikit-learn Python library [43]. As mentioned beforehand, the descriptor calculation procedure resulted in a high-dimensional feature space. This issue is a known limitation for Machine Learning (ML) algorithms. To overcome this problem, feature selection was employed to reduce the feature space to a maximum of six features that should be most important for the model’s prediction. This was carried out using the SelectFromModel class from scikit-learn, which selects features based on the importance scores calculated by a random forest regressor. Finally, the proper model was built based on the Random Forest algorithm as implemented in the scikit-learn Python library. We used a standard RF algorithm without fine-tuning:

n_estimators = 100, *, criterion = ‘squared_error’, max_depth = None, min_samples_split = 2, min_samples_leaf = 1, min_weight_fraction_leaf = 0.0, max_features = 1.0

Two models to predict surface tension and density of dental monomers were built utilizing this software. The modeling based on the dataset for regular organic compounds was also performed. The dataset on density and surface tension of general organic compounds at 298 K was extracted from the OCHEM database and was used to build two additional QSPR models.

The models were trained using a leave-one-out cross-validation approach, where each molecule was iteratively left out of the training set and predicted using the remaining molecules. The performance of the models was evaluated using the root mean squared error (RMSE) and R-squared (R^2^) metrics [44]. RF importance and Permutation importance for the models were calculated using scikit-learn. The additional models were built using CircuS descriptors. CircuS descriptors, similar to ISIDA descriptors, convert molecular fragments into non-binary vectors based on their frequency of occurrence. Unlike the other methods, they explicitly account for complex structural patterns, including closed rings, allowing for a more detailed representation of molecular substructures [45].

### 4.3. Model Interpretation

To gain a deeper understanding of the relationships between the molecular descriptors and the predicted properties, two model interpretation techniques were employed, namely: SHAP (SHapley Additive exPlanations) [46] and permutation analysis. SHAP is a technique used to explain the output of a machine learning model by assigning a value to each feature for a specific prediction, indicating its contribution to the outcome. The SHAP values represent the change in the predicted value that each feature is responsible for. In this study, the SHAP library was used to generate SHAP values for each feature in the model. Permutation analysis is a technique used to evaluate the importance of each feature in the model. The permutation analysis works by randomly permuting the values of a single feature and measuring the decrease in model performance. This process is repeated for each feature and the results are used to calculate the feature importance. In this work, the ELI5 library was used to calculate the permutation importance. To determine the atomic contributions to a predicted property according to a model based on CircuS descriptors, a fragment-based approach proposed by Marcou et al. was used [47], where the importance of each atom is calculated by analyzing the change in prediction when the atom’s fragment is perturbed. This is achieved by iteratively modifying the descriptor vector of each fragment and measuring the resulting difference in prediction, which is then assigned as a score to each involved atom. The scores are normalized between 0 and 1 and visualized using a color scheme, allowing for intuitive identification of beneficial modifications to the molecular structure. The color scheme that is used is based on two complementary colors—green and red—that are assigned to the negative and positive contributions, respectively [38].

## 5. Conclusions

The QSPR models to predict density and surface tension were built and validated. The models performed well statistically, yielding reliable predictions. The comparison between the two predicted properties shows that in most cases the presence of heteroatoms increases both the density and surface tension of monomers, but the other structural features that affect one of the analyzed properties positively have the opposite effect on the other. It is worth mentioning that the results are applicable only for the uncrosslinked phases, as the physicochemical properties noticeably change once the polymerization process begins. Obtaining a dental material with lower surface tension and lower density may reduce the risk of unsuccessful treatment, as at the same time the contraction stress and microleakage will be reduced and the adhesion strength will increase. Thus, acquiring the desired parameters of the dental composite material requires tuning the structural characteristics of the dental monomers utilizing the gained knowledge and finding the right balance of the overall proportion of monomers in the final product that is shipped to the dental offices. The next step in the research should be a multivariate model that would be able to predict multiple properties at once. This topic requires an extensive study in the future.

## Figures and Tables

**Table 1:** Comparison of different molecular representations’ impact on models’ performance.

Molecular Representation	Density		Surface Tension	
	R^2^	RMSE	R^2^	RMSE
	OCHEM datasets (5-CV)			
MFs	0.673	0.112	0.551	0.004
MDs	0.960	0.039	0.732	0.003
MFs + MDs	0.955	0.041	0.734	0.003
	Dental monomer datasets (LOOCV)			
MFs	0.763	0.042	0.494	0.003
MDs	0.641	0.052	0.768	0.002
MFs + MDs	0.737	0.044	0.758	0.002

**Table 2:** Comparison of statistical parameters of models built on combined datasets. Experimental data were gathered by the measurements carried out in the laboratory and simulated data were acquired by applying QSPR models.

Subset of Data Monomer Dataset	Density		Surface Tension	
	R^2^	RMSE	R^2^	RMSE
Experimental	0.797	0.035	<0	0.002
Experimental and simulated	0.826	0.036	0.565	0.003

**Table 3:** Descriptors and permutation analysis of the models. Higher values in both columns indicate greater influence on the model. It can be observed that the density model based on the monomers’ first three descriptors exerts almost identical influence.

Descriptor	Permutation Importance	RF Model-Based Importance
Density model based on OCHEM dataset		
AMID_h	0.210	0.126
AATS1Z	0.184	0.170
AMW	0.076	0.215
Mm	0.052	0.173
AATS2m	0.047	0.163
AATS2Z	0.046	0.152
Density model based on dental monomer dataset		
TopoPSA	0.110	0.211
AATS1Z	0.109	0.191
TopoPSA(NO)	0.109	0.153
AATS1m	0.093	0.145
ATSC1c	0.053	0.199
BCUTc-1h	0.025	0.101
Surface tension model based on OCHEM dataset		
Mv	0.690	0.357
GATS1v	0.264	0.247
VSA_EState8	0.238	0.088
nBondsM	0.192	0.078
SIC5	0.174	0.151
nHBDon	0.122	0.079
Surface tension model based on dental monomer dataset		
AATS1d	0.213	0.350
BCUTs-1h	0.177	0.210
AATS4dv	0.117	0.151
ATSC1c	0.042	0.127
MATS1dv	0.034	0.076
ATS0s	0.025	0.085

## Data Availability

The research data are available in the Electronic Supplementary Materials.

## Supplementary Materials

The following supporting information can be downloaded at: https://www.mdpi.com/article/10.3390/ijms26178283/s1.

## Appendix A. Glossary

**QSPR**—Quantitative Structure–Property Relationship. A mathematical modeling method that finds connections between the structural features of chemical compounds and their observed physicochemical properties.

**Molecular descriptors**—a set of predefined mathematical formulas connected with structural features of a chemical compound that can be used to describe a molecule in a mathematical way—in the form of a table of calculated values.

**CircuS**—CircularSubstructure 2D descriptors. Novel kind of molecular descriptor, they may be used to provide a 2D visual representation of the structural features influencing a modeled property.

**OCHEM**—Online Chemical Database. An online database containing information on physicochemical properties of numerous organic compounds and providing the possibility for the users to create novel predictive models on their own.

## Appendix B. Moreau–Broto Autocorrelation Descriptors

ATS descriptors are used to describe the distribution of a considered property along the topological structure of the analyzed compound. They are calculated as follows:

(A1) $$ATS_{k}=\frac{1}{2}\sum_{i=1}^{A}\cdot \sum_{j=1}^{A}w_{i}w_{j}\delta \left(d_{ij};k\right)$$

(A2) $$\delta \left(d_{ij};k\right)=\left\{\begin{array}{c} 1\;\;\;\;\;\;d_{ij}=k\\ 0\;\;\;\;\;\;d_{ij}\neq k \end{array}\right.$$

In the above expressions, *w* is the atomic property, *A* is the number of atoms in the molecule, *k* is the lag, *d_ij_* is the distance between *ith* and *jth* atom, and *δ* is a Kronecker delta. If lag equals 0, ATS descriptors are calculated as a sum of the squares of atomic properties:

(A3) $$ATS_{0}=\sum_{i=1}^{A}w_{i}^{2}$$

ATS descriptors can be averaged, which is achieved by dividing them by the number of vertex pairs at the *k* lag, which is a sum of Kronecker delta (Δ*k*), as shown below in Equation (A4). Averaged ATS (AATS) descriptors calculated this way are independent of the influence of molecular size.

(A4) $$AATS_{k}=\frac{ATS_{k}}{\Delta k}$$

Another modification of ATS descriptors are the centered ATS descriptors (ATSCs), which are calculated by subtracting the average property value calculated for the whole molecule from each individual property value. ATSC descriptors are calculated almost in the same way as ATS descriptors, but the property *w* is substituted by *w_c_* parameter, which is calculated as described above and shown in Equations (A5) and (A6). Operating on the centered descriptors assures that they are not correlated, thus leading to better statistical parameters of the models [26,48,49].

(A5) $$ATSC_{k}=\frac{1}{2}\sum_{i=1}^{A}\cdot \sum_{j=1}^{A}w_{ci}w_{cj}\delta \left(d_{ij};k\right)$$

(A6) $$w_{c}=w-\bar{w}$$

There are also indexes of spatial autocorrelation that take into consideration both averaged and centered data at the same time. One of them is the Moran coefficient (*I_k_*), which is used to calculate MATS descriptors. Their value ranges from −1 to +1 and strong negative autocorrelation to strong positive autocorrelation correspond to that range, respectively. The Moran coefficient is calculated as follows [26,50]:

(A7) $$AATSC_{k}=\frac{1}{2\Delta k}\sum_{i=1}^{A}\cdot \sum_{j=1}^{A}w_{ci}w_{cj}\delta \left(d_{ij};k\right)$$

(A8) $$I_{k}=MATS_{k}=\frac{AATSC_{k}}{\frac{1}{A}\sum_{i=1}^{A}w_{c}^{2}}$$

The last subcategory of ATS descriptors in the study are the GATS descriptors that are calculated using Geary’s coefficient (*c_k_*). Geary’s positive autocorrelation corresponds to values of GATS descriptors in the range < 0;1), a value of 1 indicates no correlation and every value over 1 means that the correlation in negative. When areas of similar value of analyzed property occur in the molecule alternately between the areas of different value, the Geary’s autocorrelation is negative. On the contrary, if they are formed in vicinity of each other, the correlation is positive. Equation (A9) shows how the GATS descriptors are calculated [26,51].

(A9) $$c_{k}=GATS_{k}=\frac{\frac{1}{2\Delta k}\sum_{i=1}^{A}\cdot \sum_{j=1}^{A}{\left(w_{i}-w_{j}\right)}^{2}\delta \left(d_{ij};k\right)}{\frac{1}{A-1}\sum_{i=1}^{A}w_{c}^{2}}$$

The variables in Equations (A7)–(A9) are the same as were described in Equations (A1)–(A6) above, therefore they will not be listed again.
