# Supplementary material for: In Search of the Perfect Composite Material—A Chemoinformatics Approach Towards the Easier Handling of Dental Materials
Source: Int J Mol Sci. 2025 Aug 26;26(17):8283. doi: 10.3390/ijms26178283 (PMC12428768; doi:10.3390/ijms26178283)
Supplement: Supplementary file 1 [file ijms-26-08283-s001.zip › ESI - Table S3 and Figure S1.pdf]

Supplementary Information – Table S3 and Figure S1

In Search of the Perfect Composite Material—A Chemoinformatics Approach Towards the Easier Handling of Dental Materials

Joachim Eichenlaub [1,2,*], Karol Baran [1], Kamil Urbański [3], Marlena Robakowska [4], Jolanta Kalinowska [3], Bogna Racka-Pilszak [3] and Adam Kloskowski [1]

[1] Department of Physical Chemistry, Faculty of Chemistry, Gdansk University of Technology, Narutowicza 11/12, 80-233 Gdansk, Poland; adam.kloskowski@pg.edu.pl (A.K.)
[2] Department of Dental Prosthetics, Medical University of Gdansk, Elizy Orzeszkowej 18, 80-208 Gdansk, Poland
[3] Division of Orthodontics, Faculty of Medicine, Medical University of Gdansk, Aleja Zwycięstwa 42c, 80-210 Gdansk, Poland; jolanta.kalinowska@gumed.edu.pl (J.K.); bogna.racka-pilszak@gumed.edu.pl (B.R.-P.)
[4] Department of Public Health and Social Medicine, Medical University of Gdansk, Debinki 7, 80-210 Gdansk, Poland; marlena.robakowska@gumed.edu.pl
* Correspondence: joachim.eichenlaub@gumed.edu.pl

Table S3. Representation of selected monomers using CircuS descriptors.

Nr	Dental monomer	Smiles code	Structure	Surface	Density
1	2-Ethoxyethyl methacrylate	CCOCCOC(=O)C(C)=C	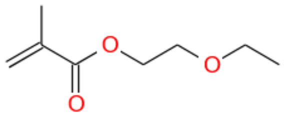	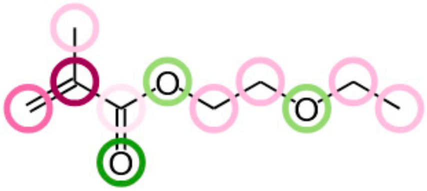	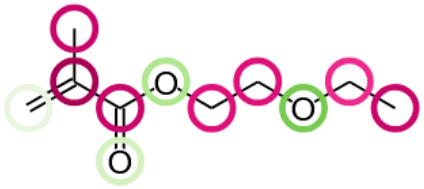
2	2-Hydroxyethyl acrylate	OCCOC(=O)C=C	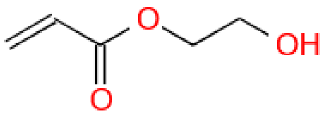	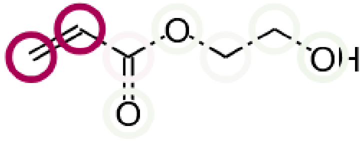	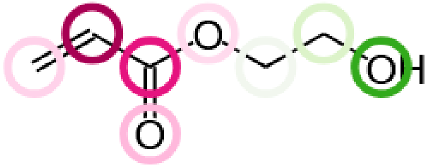

3	2-Hydroxyethyl methacrylate	CC(=C)C(=O)OCCO	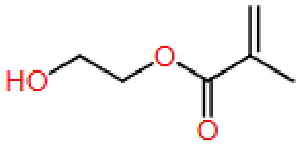	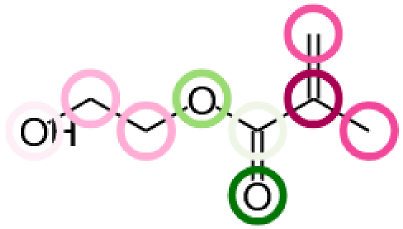	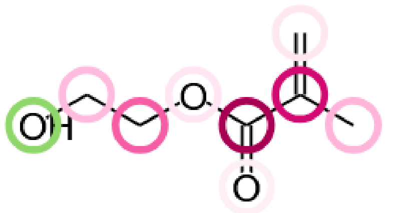
4	2-Hydroxypropyl acrylate	CC(O)COC(=O)C=C	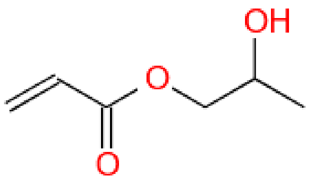	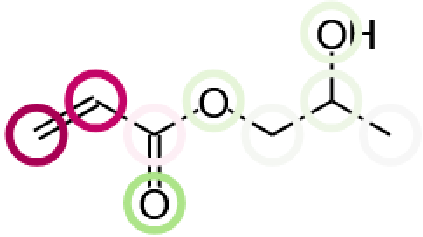	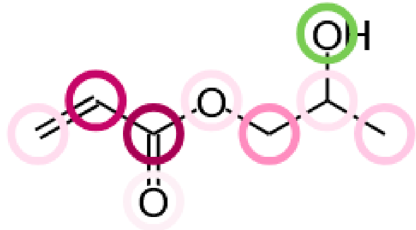
5	2-Hydroxypropyl methacrylate	CC(O)COC(=O)C(C)=C	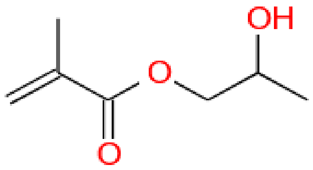	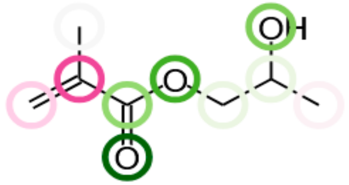	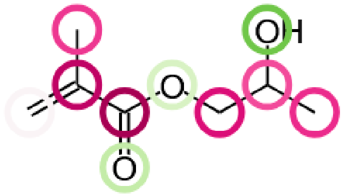
6	2-Methoxyethyl methacrylate	COCCOC(=O)C(C)=C	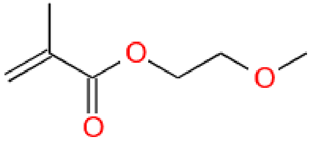	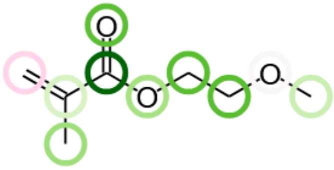	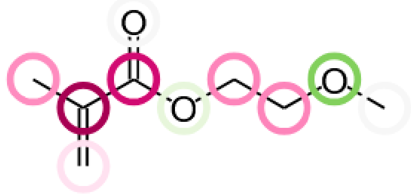
7	2-Phenoxyethyl methacrylate	CC(=C)C(=O)OCCOc1ccccc 1	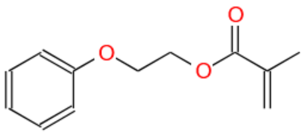	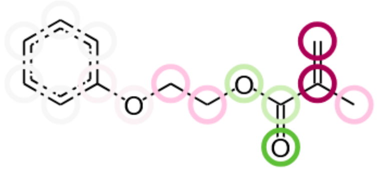	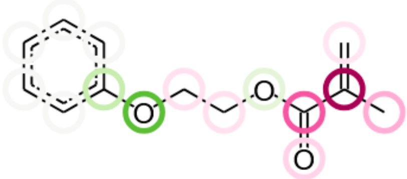

8	Acrylamide	C=CC(=O)N	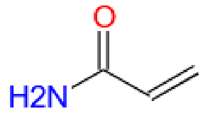	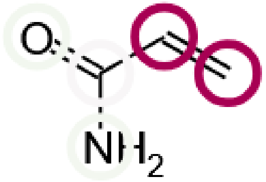	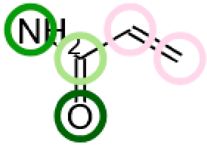
9	Allyl methacrylate	CC(=C)C(=O)OCC=C	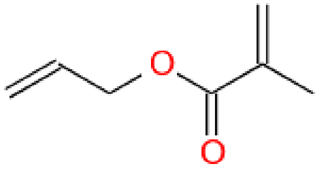	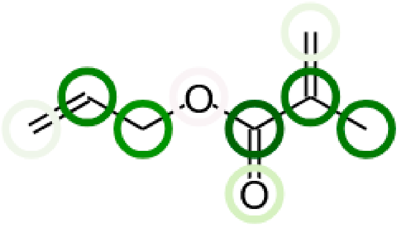	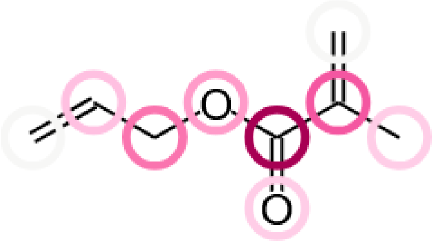
10	Benzyl methacrylate	CC(=C)C(=O)OCc1ccccc1	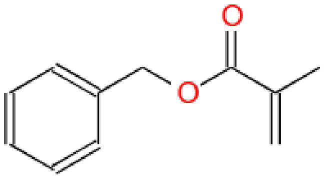	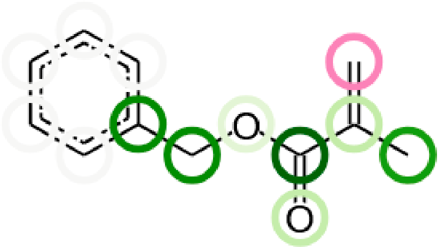	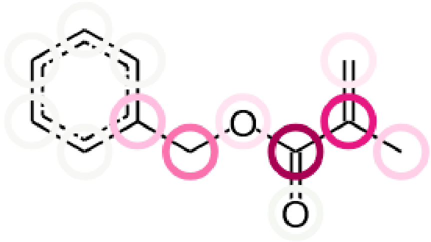
11	Dimethylaminoethyl methacrylate	CN(C)CCOC(=O)C(C)=C	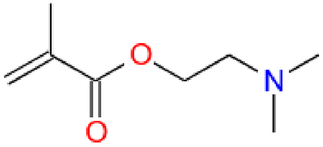	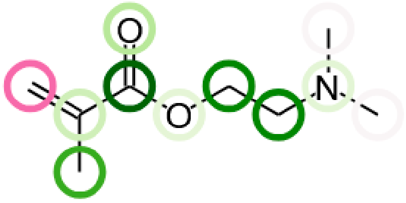	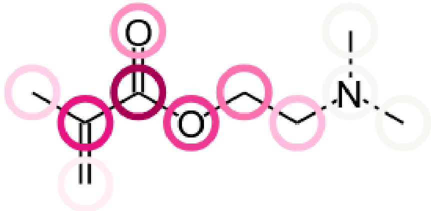
12	Ethyl acrylate	CCOC(=O)C=C	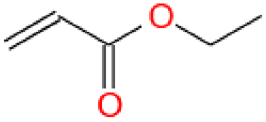	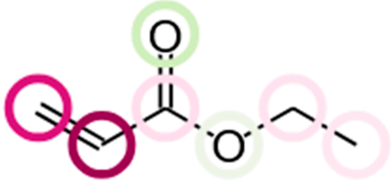	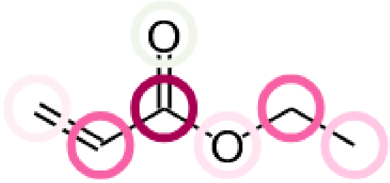

13	Ethyl methacrylate	CCOC(=O)C(C)=C	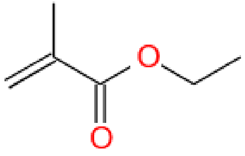	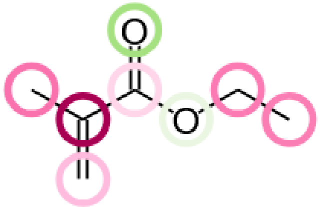	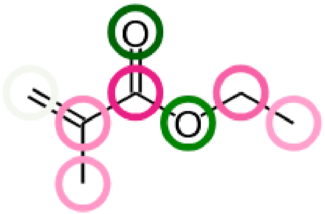
14	Ethyleneglycol dimethacrylate	CC(=C)C(=O)OCCOC(=O)C(=C)C	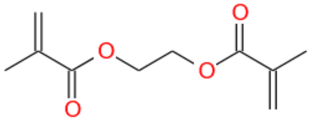	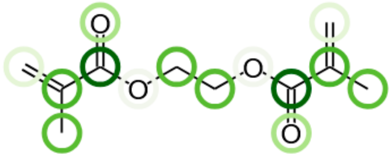	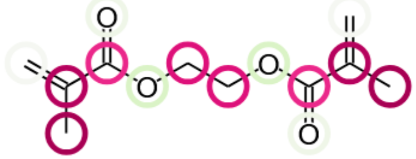
15	Hexyl acrylate	CCCCCCOC(=O)C=C	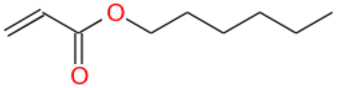	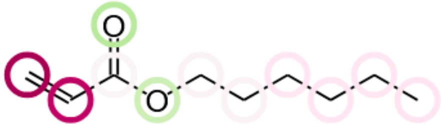	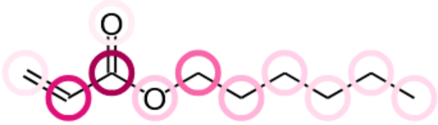
16	Isobutyl acrylate	CC(C)COC(=O)C=C	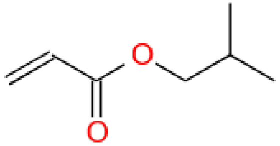	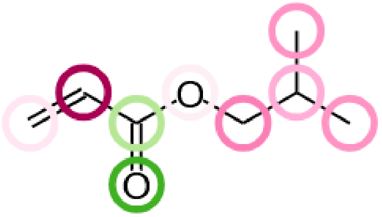	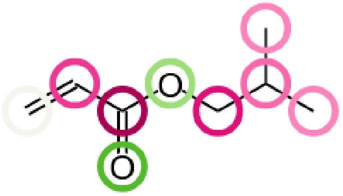
17	Isobutyl methacrylate	CC(C)COC(=O)C(=C)C	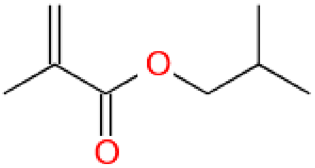	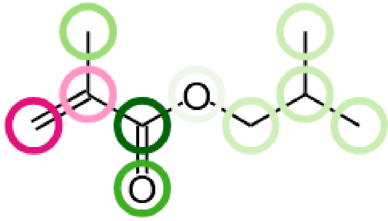	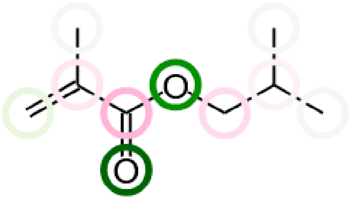

18	Methacrylic acid	CC(=C)C(=O)O	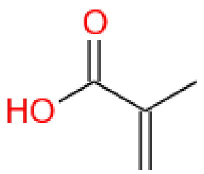	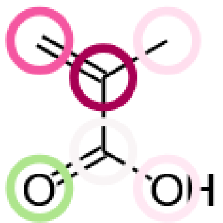	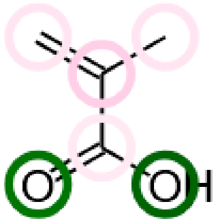
19	Methyl acrylate	COC(=O)C=C	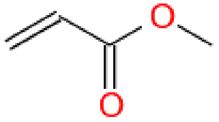	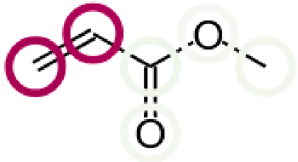	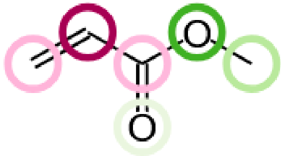
20	Methyl methacrylate	COC(=O)C(C)=C	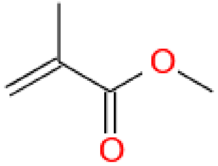	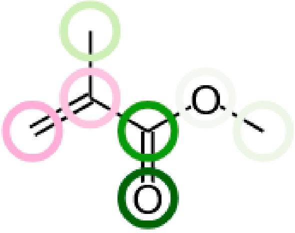	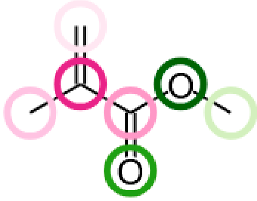
21	n-Butyl acrylate	CCCCOC(=O)C=C	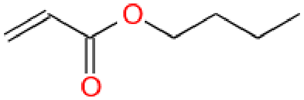	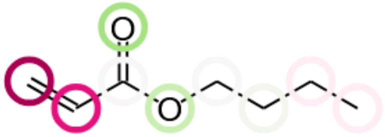	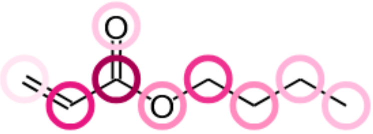
22	n-Butyl methacrylate	CCCCOC(=O)C(C)=C	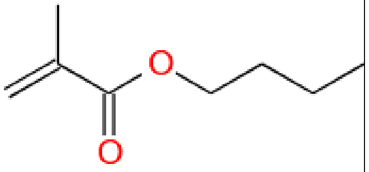	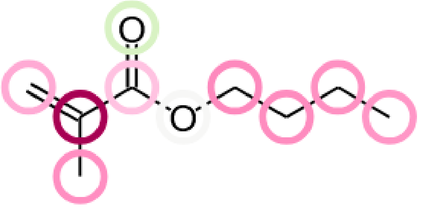	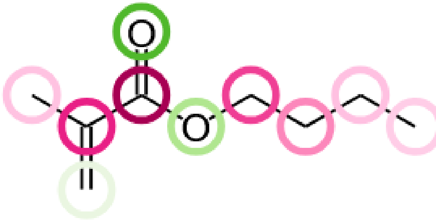

23	n-Propyl acrylate	CCCOC(=O)C=C	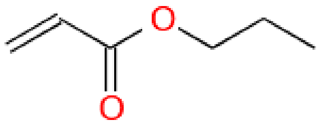	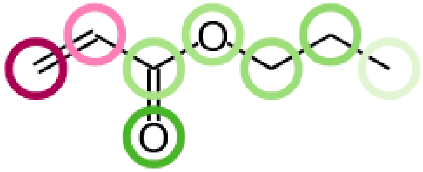	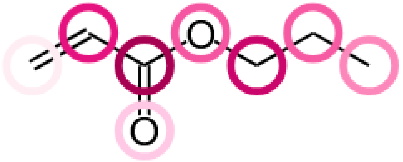
24	n-Propyl methacrylate	CCCOC(=O)C(C)=C	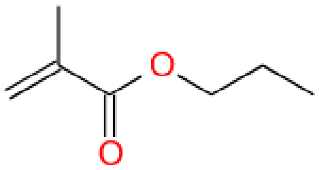	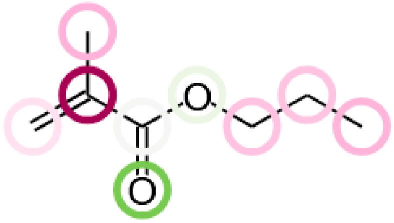	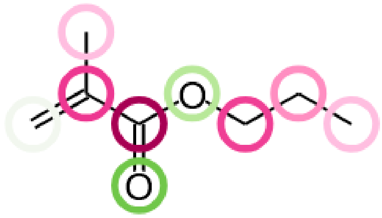
25	Tetrahydrofurfuryl methacrylate	CC(=C)C(=O)OCC1CCCO1	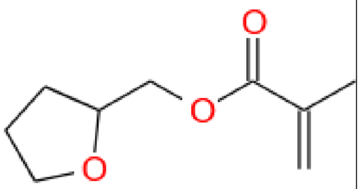	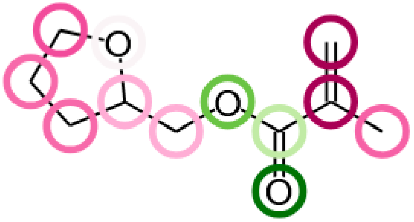	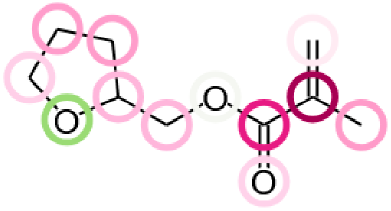

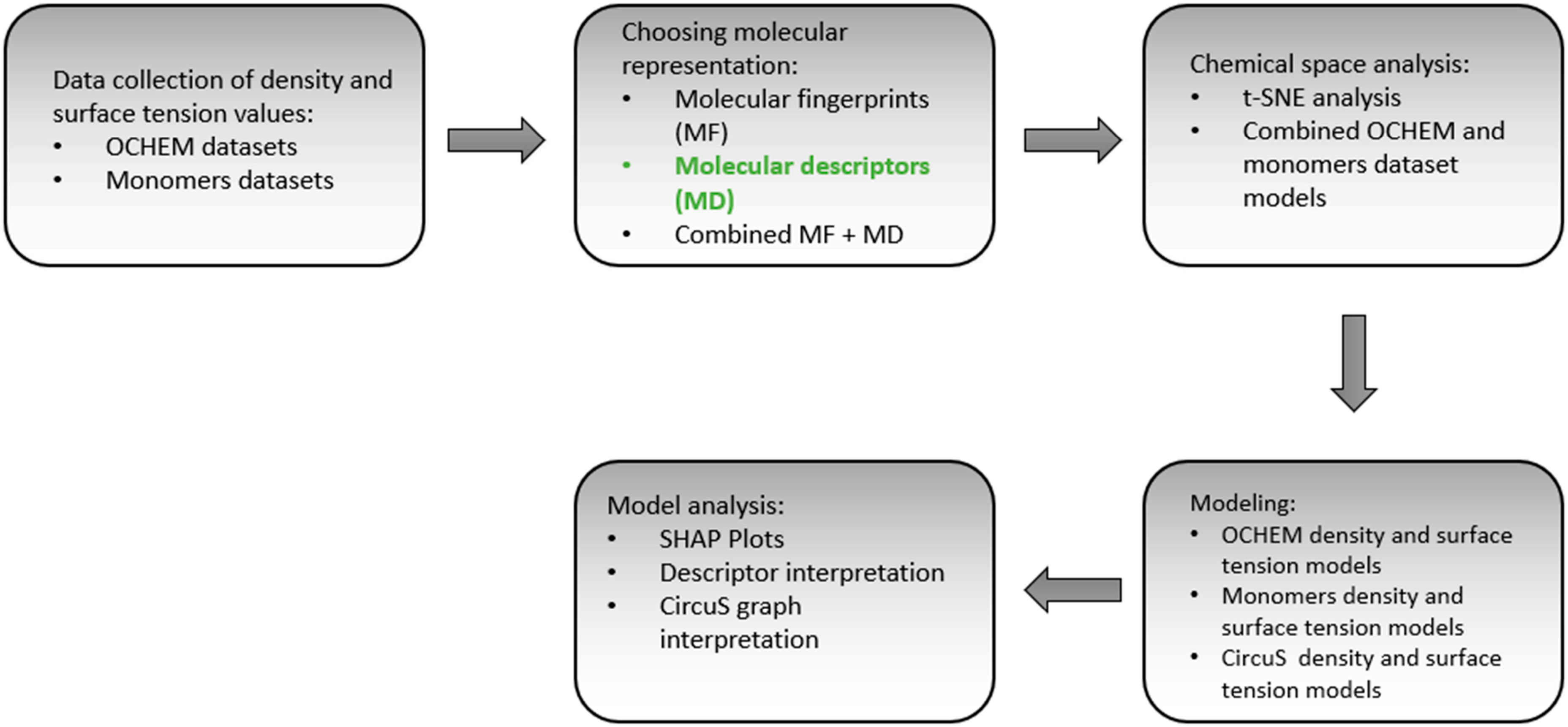

Figure S1. Workflow of the study.
